# Perspectives in Melanoma: meeting report from the Melanoma Bridge (December 3rd–5th, 2020, Italy)

**DOI:** 10.1186/s12967-021-02951-x

**Published:** 2021-06-30

**Authors:** Paolo A. Ascierto, Christian Blank, Reinhard Dummer, Marc S. Ernstoff, Soldano Ferrone, Bernard A. Fox, Thomas F. Gajewski, Claus Garbe, Patrick Hwu, Pawel Kalinski, Michelle Krogsgaard, Roger S. Lo, Jason J. Luke, Bart Neyns, Michael A. Postow, Sergio A. Quezada, Michele W. L. Teng, Giorgio Trinchieri, Alessandro Testori, Corrado Caracò, Iman Osman, Igor Puzanov, Magdalena Thurin

**Affiliations:** 1 Department of Melanoma, Cancer Immunotherapy and Innovative Therapy, Instituto Nazionale Tumori IRCCS “Fondazione G. Pascale”, Naples, Italy; 2 Netherlands Cancer Institute, Amsterdam, The Netherlands; 3 Department of Dermatology, University of Zurich Hospital, Zurich, Switzerland; 4 Developmental Therapeutics Program, Division of Cancer Therapy & Diagnosis, NCI, NIH, Bethesda, MD USA; 5 Department of Surgery Massachusetts General Hospital, Harvard Medical School, Boston, MA USA; 6 Earle A. Chiles Research Institute, Robert W. Franz Cancer Center, Providence Cancer Institute, Portland, OR USA; 7 Department of Pathology and Department of Medicine (Section of Hematology/Oncology), University of Chicago, Chicago, IL USA; 8 Center for Dermato-Oncology, University-Department of Dermatology, Tuebingen, Germany; 9 Moffitt Cancer Center, Tampa, FL USA; 10 Cancer Vaccine and Dendritic Cell Therapies, Center for Immunotherapy, Roswell Park Comprehensive Cancer Center, Developmental Therapeutics, Buffalo, NY USA; 11 Medical School, New York University Langone, New York, NY USA; 12 Jonsson Comprehensive Cancer Center David Geffen School of Medicine at UCLA, Los Angeles, CA USA; 13 Cancer Immunotherapeutic Center of UPMC Hillman Cancer Center, University of Pittsburgh, Pittsburgh, PA USA; 14 Medical Oncology, Universitair Ziekenhuis Brussel, Brussels, Belgium; 15 Memorial Sloan Kettering Cancer Center, New York, NY USA; 16 Weill Cornell Medical College, New York, NY USA; 17 Cancer Immunology Unit, Research Department of Hematology, University College London Cancer Institute, London, UK; 18 QIMR Berghofer Medical Research Institute, Herston, QLD Australia; 19 Laboratory of Integrative Cancer Immunology (LICI), Center for Cancer Research, NCI, NIH, Bethesda, MD USA; 20 Image Rigenerative Clinic-Skin Oncology Division, Milan, Italy; 21 Chairman Surgical Subgroup EORTC Melanoma Group Brussels, Brussels, Belgium; 22 Division of Surgery of Melanoma and Skin Cancer, Istituto Nazionale Tumori “Fondazione Pascale” IRCCS, Naples, Italy; 23 New York University Langone Medical Center, New York, NY USA; 24 Department of Medicine, Roswell Park Comprehensive Cancer Center, Buffalo, NY USA; 25 Cancer Diagnosis Program, Division of Cancer Treatment and Diagnosis, NCI, NIH, Rockville, MD USA

**Keywords:** Melanoma, Immunotherapy, Anti-PD-1, Anti-CTLA-4, Target therapy, Biomarkers, BRAF inhibitor, MEK inhibitor, Adjuvant, Neoadjuvant, Combination strategies

## Abstract

Advances in immune checkpoint therapy and targeted therapy have led to improvement in overall survival for patients with advanced melanoma. Single agent checkpoint PD-1 blockade and combination with BRAF/MEK targeted therapy demonstrated benefit in overall survival (OS). Superior response rates have been demonstrated with combined PD-1/CTLA-4 blockade, with a significant OS benefit compared with single-agent PD-1 blockade. Despite the progress in diagnosis of melanocytic lesions, correct classification of patients, selection of appropriate adjuvant and systemic therapies, and prediction of response to therapy remain real challenges in melanoma. Improved understanding of the tumor microenvironment, tumor immunity and response to therapy has prompted extensive translational and clinical research in melanoma. Development of novel biomarker platforms may help to improve diagnostics and predictive accuracy for selection of patients for specific treatment. There is a growing evidence that genomic and immune features of pre-treatment tumor biopsies may correlate with response in patients with melanoma and other cancers but they have yet to be fully characterized and implemented clinically. Overall, the progress in melanoma therapeutics and translational research will help to optimize treatment regimens to overcome resistance and develop robust biomarkers to guide clinical decision-making. During the Melanoma Bridge meeting (December 3rd–5th, 2020, Italy) we reviewed the currently approved systemic and local therapies for advanced melanoma and discussed novel biomarker strategies and advances in precision medicine.

## Introduction

The treatment of metastatic melanoma has undergone a dramatic transformation over the past decade with the advent of molecular targeted therapy and immunotherapy. Therapeutic approaches targeting mutated BRAF 600 mutations (e.g. vemurafenib, dabrafenib, and encorafenib) are Food and Drug Administration (FDA)-approved drugs that have been developed as inhibitors of BRAF V600 mutations. While monotherapy with BRAF inhibitors shows good efficacy against BRAF-mutant melanomas, patients can easily develop resistance. As a result, combination therapy with BRAF and MEK inhibitors, including trametinib and cobimetinib, has become standard to inhibit melanoma growth. Direct targeting of NRAS oncogene is difficult, and therapies focused on targeting its downstream signals including MEK inhibitors have been identified as potential therapy for NRAS mutants. Despite the success of target therapies, the therapy failure in many patients suggests that much remains to be learned about the mechanism or response and resistance to these drugs.

Immune checkpoint inhibitors represent a novel class of drugs that have increasingly been used in melanoma therapy. Major advances in targeting the immune evasion phase of tumors have been achieved using drugs that block the inhibitory check points that regulate the immune system, such as programmed death 1 (PD-1) and cytotoxic T-lymphocyte-associated antigen (CTLA-4) and other T cell inhibitory and activating receptors. Dual immune checkpoint blockade with ipilimumab and nivolumab enhances response rates compared with single agent ipilimumab or nivolumab in patients with metastatic melanoma (response rate, 58%), and both nivolumab containing arms demonstrated superior overall survival (OS) compared with single agent ipilimumab.

Several approaches in immunotherapy that include monoclonal antibodies, vaccines, biochemotherapy, and the transfer of adoptive T cells, natural killer (NK) cells, dendritic cells and bispecific antibodies are currently under investigation for the treatment of melanoma. These treatments have the same goal as drugs that are already used to stimulate the proliferation of T lymphocytes in order to destroy tumor cells. Another form of immunotherapy active in cutaneous melanoma is the injectable agent talimogene laherparepvec (T-VEC).

While immunotherapy shows promising potential, predicting therapeutic response has proven difficult. Programmed death ligand-1 (PD-L1) has been studied as a potential marker to predict response to immunotherapy PD-L1. PD-L1 expression, a T cell-inflamed tumor microenvironment, and tumor mutation burden (TMB) have been shown to be associated with improved response rates to checkpoint blockade therapy; however, absence of expression did not rule out a chance for response to combined or monotherapy.

Timely diagnosis of melanoma is critical for effective therapy, but histopathologic diagnosis can frequently present significant challenges to this goal. Diagnostic and therapeutic molecular markers have been increasingly used to assist in histopathological assessment of especially of histologically challenging cases. Detecting molecular markers such as genetic alterations has emerged as an innovative diagnostic and predictive biomarkers that guides therapeutic decisions. Biomarkers that are represented by gene mutations in the mitogen-activated protein kinase (MAPK) and PI3K/AKT signaling pathways (e.g. BRAF, NRAS, MEK, ERK) are the most common genes affected in cutaneous melanoma. The hallmarks mutations in uveal melanoma (e.g. GNAQ/GNA11, BAP, SF3B1, EIF1AX) and acral melanoma (e.g. KIT) have also been identified. These markers are not only helpful for diagnosing melanoma, but also in distinguishing certain subtypes that can guide the selection of treatment and development of novel targeted therapies.

Despite the dramatic improvement in clinical outcomes over the past decade owing to immunotherapy and targeted therapy in melanoma, not all patients respond to approved systemic therapies. Extensive preclinical, translational, and clinical research is ongoing to better understand the mechanisms of response and resistance to current therapies, develop rational next-generation treatments (and combinations), and develop better models of melanoma that will support further preclinical and translational research.

## Melanoma as a model system—session

### Targeting the microbiota in melanoma anti-PD1 therapy

The composition of the gut microbiome determines the efficacy of cancer therapy by modulating the anti-tumor immune response through the training of infiltrating myeloid and antigen-presenting cells in the tumor. This has been shown in murine models, in which mice with distinct gut microbiota profiles exhibited differential tumor growth and differences in response to PD-1 blockade. These differences could be eliminated by cohousing of animals, indicating it could be transmitted. Fecal microbiota transplant (FMT) together with anti-PD-1 therapy resulted in nearly full tumor rejection in mice with melanoma.

The influence of the microbiome on the effect of immune checkpoint blockade has also been shown in several clinical studies. In one study of melanoma patients treated with PD-1 inhibitors, significant differences were observed in the gut microbiome between responders and non-responders, with responders having higher diversity and more *Ruminococcaceae* bacteria [1]. These patients also had enhanced systemic and antitumor immunity. In another study, a significant association was observed between microbiome composition and clinical response to PD-1 blockade in melanoma patients, with *Bifidobacterium longum*,* Collinsella aerofaciens*, and *Enterococcus faecium* all more abundant in responders [2].

One issue is that different studies have identified a wide variety of different bacterial species that are associated with response. In a meta-analysis of several studies, metagenomics identified *Ruminococcaceae*,* Lachnospiraceae*, and *Bifidobacteriaceae* as being associated with a response to anti-PD-1 treatment, while *Bacteroidaceae* were generally associated with a lack of response.

The potential role of FMT is being assessed in a phase II trial at the University of Pittsburgh, in which fecal samples obtained from long-term PD-1 responders is combined with additional anti-PD-1 treatment in melanoma patients who previously failed to respond to PD-1 blockade [3]. To date, 16 anti-PD-1 refractory patients have received FMT from PD-1 responders, with one complete response, two partial responses and three with stable disease. Consistent with previous observations, responders tended to have higher frequency of *Ruminococcaceae, Lachnospiraceae*, and *Bifidobacteriaceae*, while *Bacteroidaceae* were more frequent in patients with disease progression. FMT induced a rapid and persistent alteration and instability in the microbiome composition, although each patient generally maintained a distinct microbiome based on their existing taxa before receiving FMT. The majority of taxa that were present in the donor but not the recipient colonized the donor gut and were persistent unless the patient was treated with antibiotics. Overall, the microbiota composition after FMT reflected colonization with the donor-specific taxa but perturbation of the microbiome resulted in altered abundance of different taxa of both donor and recipient origin.

FMT in anti-PD-1 refractory melanoma is most likely to induce a response in patients with the immunological potential to respond but with an unfavorable microbiota that can be corrected. However, anti-PD-1 refractory patients may fail FMT for various reasons. These may include the absence of an adequate immunological response regardless of microbiota composition, the FMT lacking the taxa needed to improve anti-PD-1 response, or the FMT failing to induce a perturbation of the microbiome that favors a response, due either to technical reasons or possibly because of incompatibility between donor and recipient microbiome.

### Prediction of response to checkpoint inhibition: is there a simple but not simplistic way?

A subset of patients with metastatic melanoma have durable responses to immunotherapy, while others develop potentially serious immune-related adverse events. Reliable biomarkers that can predict response to immune checkpoint inhibition are needed but remain elusive. PD-L1 expression, and TMB are used in the clinic but have limitations, as do other baseline characteristics that have been proposed, e.g. lactate dehydrogenase (LDH) and ECOG performance status. Active areas of research to predict immune checkpoint inhibitor response include biomarkers in the blood and microbiome, genomic profiling of the T cell regulome, auto-antibody signatures for immune-related toxicity and microRNA (miRNA) profiling.

Another possible approach is to integrate machine learning technology on histology specimens with clinical data to predict immune checkpoint inhibitor response. Previously, our group developed a deep convolutional neural network pipeline that could discriminate between malignant and normal lung tissue. In addition, the network was trained to accurately predict the most frequently mutated genes in lung tumors including STK11, EGFR, FAT1, SETBP1, KRAS and TP53 [4]. This machine learning framework was then adapted to whole slide image analysis of tissue from patients with metastatic melanoma who had lymph node and/or subcutaneous tissue resected before first-line anti-CTLA-4 and/or anti-PD-1 therapy [5].

Google Inception v3 was used as a foundation architecture for computational image analysis. Using hematoxylin and eosin-stained slides, a neural network segmentation classifier was trained to identify the tumor from the surrounding microenvironment. Because the dataset included lymph node and subcutaneous tissue sections, the classifier was trained to identify lymphocyte clusters and connective tissue in addition to tumor compartments. This segmentation classifier distinguished regions of interest with high accuracy. A response classifier was then trained to identify whether patients responded to checkpoint blockade or were resistant and progressed. A logistic regression classifier that combined neural network output with clinical and demographic variables (ECOG performance status) augmented the prediction accuracy. Class activation mapping revealed that cell nuclei played an important role in the decision to classify samples as progressive disease or response, with more and larger nuclei associated with a prediction of progression. Based on these findings, machine learning on metastatic melanoma tissue histology shows potential for predicting immunotherapy response, especially if integrated with clinical data.

Another approach is to assess the relationship between body mass index (BMI) and outcomes. Studies have suggested a link between BMI and response to checkpoint blockade, including the counterintuitive phenomenon in which a high BMI seems to confer a survival benefit [6]. However, there are substantial discordances in these data. For example, we found that patients who were overweight or obese has similar progression-free survival (PFS) as patients with normal BMI [7]. However, there was a moderate but non-significant association between higher BMI and better PFS in patients receiving first-line treatment. In addition, a significant survival benefit was observed in overweight and obese patients receiving combination immunotherapy, whereas this was not seen in patients who received anti-PD-1 or anti-CTLA-4 monotherapy.

One possibility is that static measurements do not consider changes in body weight and nutritional intake over time. Moreover, in some patients a lower BMI might be associated with disease progression. To investigate this, we tested the association between BMI changes before the start of immunotherapy and treatment outcomes in patients with melanoma, lung cancer or other cancers [8]. A pretreatment decrease in BMI and low baseline prognostic nutritional index were associated with worse outcomes, including PFS and OS. However, baseline BMI category was not significantly associated with any treatment outcomes, indicating that dynamic BMI changes rather than static assessments correlate with treatment response.

### Immunotherapy-induced anti-cancer responses

The anti-cancer immune response involves B and T cell responses against the cancer surfaceome, which includes non-mutated shared antigens, mutated epitopes, and the extracellular domains of transmembrane proteins, as well as intracellular proteins released from tumor cells. There is considerable evidence that shared antigens are important, and genes that are upregulated or amplified in cancer and are associated with worse survival outcomes are potential therapeutic targets. An example of an overexpressed normal gene is vestigial-like (VGLL)-1 that is a cancer-placenta antigen. VGLL-1-specific cytotoxic T lymphocytes (CTLs) can recognize and kill human leukocyte antigen (HLA)-matched allogeneic tumor cell lines derived from different cancers in an antigen-specific manner, indicating that VGLL-1 may constitute an immunotherapeutic target in multiple cancer types [9].

Peptides that are presented on tumor human leukocyte antigen (HLA) molecules are mostly derived from short-lived proteins (SLiPs) and defective ribosomal products (DRiPs) bound to HLA and transported to the cell surface. However, these rapidly degraded products are less available for cross-presentation by antigen-presenting cells and as such are not typical targets of the immune system.

Blocking proteasomal degradation leads to stabilization of DRiPs and SLiPs and the formation of autophagosomes that contain not only DRiPs/SLiPs, but also damage-associated molecular patterns (DAMPs) and chaperone molecules that facilitate cross-presentation. These autophagosomes can be harvested by membrane disruption and fractionation to create the DRibbles vaccine product. The first allogeneic human DRibbles vaccine, DPV-001, was derived from autophagosome products of two non-small-cell lung cancer (NSCLC) cell lines, one of mixed histology and one from an adenocarcinoma [10]. DPV-001 contains multiple toll-like receptor agonists and > 130 potential NSCLC antigens. In a phase II trial, patients with stage III NSCLC received cyclophosphamide induction therapy, before being randomized to DPV-001 alone, or in combination with granulocyte–macrophage colony-stimulating factor or imiquimod. Patients receiving DPV-001 had a significant increase in total (CD4 and CD8) T cells versus controls and the increase in CD4 T cells was similar to that seen in patients receiving ipilimumab.

Antibody responses to over-expressed antigens were detected as ‘waves’ with possible co-coordination of B cell and T cell responses after vaccination. T cell contraction, a natural component of the T cell response to antigens, may be responsible for this anomaly. Co-stimulation with T cell agonists, such as OX40, GITR or 4-1BB, may augment vaccine-induced T cell expansion, maintenance, and function. In preclinical models, DRibbles vaccine and anti-OX40 co-stimulation led to tumor regression and improved survival in a breast cancer murine model [11]. Similarly, anti-GITR and anti-PD-1 antibodies in combination with DRibbles vaccine resulted in better survival in a pancreatic cancer model [12]. Three triplet immunotherapy trials are now ongoing; a phase 1b trial of multivalent autophagosome vaccine with or without OX40 antagonist with nivolumab in patients with triple-negative breast cancer (NCT02737475), a phase 1b trial of multivalent autophagosome vaccine with or without GITR antagonist with anti-PD-1 in patients with head or neck squamous cell carcinoma (NCT04470024), and a trial of neoadjuvant/adjuvant GVAX pancreas vaccine with or without nivolumab and urelumab in patients with surgically resectable pancreatic cancer (NCT02451982).

### The role of CD39 in melanoma

The ATP-adenosine pathway is a key modulator of innate and adaptive immunity within the tumor microenvironment (TME). CD39 is the rate-limiting enzyme in the conversion of ATP to immunomodulatory adenosine. Extracellular adenosine in the TME favors escape from antitumor immunity and tumor progression. CD39 and adenosine receptors are upregulated in response to stimuli such as hypoxia, tissue damage and remodeling, and chronic inflammation. High levels of CD39 have been reported in various tumors and CD39 as a therapeutic target has been an active area of investigation.

CD39 blockade with the broad ectonucleotidase inhibitor sodium polyoxotungstate (POM-1) has shown improved antitumor immunity and decreased metastatic burden in preclinical models. However, concerns over its lack of specificity, potential toxicity and limited therapeutic half-life have hindered its clinical development. More recently, anti-CD39 antibodies have been generated. A novel anti-mouse CD39 antibody, which specifically binds to CD39-expressing cells and potently inhibits CD39 ATPase activity in vitro has demonstrated potent activity against MC38 colon adenocarcinoma tumors [13]. Anti-CD39 monotherapy was as potent as anti-PD-1 antibody and more effective than anti-CD73 antibodies and adenosine A2 receptor antagonists in this model. Anti-CD39 was also shown to sensitize anti-PD-1 resistant tumors by increasing CD8+ T cell infiltration. Inhibition of CD39 enzymatic function led to an accumulation of extracellular ATP, which in turn led to an activation of myeloid cells via ATP receptor P2X7. The antitumor activity of anti-CD39 required CD39 and P2X7 co-expression on intratumor myeloid subsets and active interleukin (IL)-18 release to facilitate expansion of intratumor effector T cells.

Targeting CD39 also suppresses experimental lung carcinoma metastases. The antimetastatic activity of anti-CD39 was NK cell and interferon (IFN)-γ dependent, and anti-CD39 enhanced IFN-γ production and CD107a expression in lung-infiltrating NK cells following tumor challenge [14]. Efficacy of anti-CD39 required enzymatic blockade but not FcR engagement. Anti-metastatic activity was dependent on the P2X7-NLRP3 inflammasome pathway. Anti-CD39 also combined with other NK cell activating agents to suppress experimental lung metastases.

An anti-human CD39 antibody enhanced CD4+ and CD8+ T cell proliferation and Th1 cytokine secretion [IFN-γ, tumor necrosis factor (TNF)-α and interleukin (IL)-2] in vitro. Anti-human CD39 antibody also enriched intratumoral human CD8+ T cells and suppressed human B-cell lymphoma following autologous Epstein-Barr virus-specific T cell transfer. First-in-human trials of the anti-CD39 antibody in patients with advanced cancer have recently been initiated.

### Adrenergic receptors: a non-canonical immune checkpoint?

The sympathetic nervous system has a role in regulating immune responses. The tumor is innervated by the sympathetic nervous system and, in response to stress, these nerves secrete norepinephrine. Chronic stress may be detrimental because it suppresses effector immune cells while activating immunosuppressive cells. β-adrenergic receptors are expressed by many cell types in the TME and β-adrenergic receptor signaling acts through multiple mechanisms to promote tumor survival, growth, and metastasis [15].

In murine models, β-adrenergic receptor antagonists (i.e., β-blockers) have been shown to improve the antitumor immune response. Chronic adrenergic signaling in mice exposed to stress promotes tumor growth. Reducing β-adrenergic receptor signaling through the use of the β-blocker propranolol facilitated conversion of tumors to an immunologically active TME and was associated with a significantly increased efficacy of anti-PD-1 checkpoint blockade [16]. Retrospective studies have also suggested that incidental use of β-blockers is associated with better survival outcomes in cancer patients. In patients with metastatic melanoma who received immunotherapy, OS was improved by use of pan-β-blockers [17]. In a small prospective study of patients with stage IB-IIIA cutaneous melanoma, those willing to take propranolol as an adjuvant treatment had an approximately 80% reduction in risk of recurrence versus those who did not take propranolol [18].

The potential of β-blockers to improve response to checkpoint blockade has been further explored in a phase I trial of propranolol and pembrolizumab in combination in patients with locally advanced and metastatic melanoma [19]. Nine patients received increasing doses of propranolol in combination with pembrolizumab 200 mg every 3 weeks. No dose-limiting toxicities were observed, and the most frequent treatment-related adverse events were rash, fatigue, and vitiligo. Objective response rate (ORR) was 78%. Perceived Stress Score decreased over time, but baseline score did not predict response. Responders tended to have a higher ratio of inflammatory (T helper cells and CTLs) to regulatory cells [myeloid-derived suppressor cells (MDSCs) and regulatory T cells (Tregs)] in the TME at baseline, suggesting this may predict for outcome. Responses seen in this small study support the potential for pan-β-adrenergic blockade to synergize with anti-PD-1 inhibition. A phase II multicenter study is currently underway.

### Escape mechanisms in melanoma

HLA class 1 antigen-processing machinery (APM) defects are frequently present in malignant tumors. Mutations in the HLA class I genes themselves, abnormalities in their regulation and/or defects in HLA class I-dependent antigen processing can underlie HLA class I downregulation. Beta 2 Microglobulin (β2m) mutations inhibit HLA class I heavy chain-β2m-peptide trimolecular complex formation on melanoma cells. However, structural defects in HLA class I heavy chain, β2m, and HLA class I APM components are caused by mutations in only a low percentage of malignancies, at most 25% of the total defects. Multiple regulatory mechanisms can be involved but the most frequent cause of HLA class I APM defects in malignancies is represented by abnormalities in epigenetic pathways. This can involve reduced HLA class I APM components chromatin accessibility, methylation of the promoter regions of HLA class I APM components by DNA methyltransferases, lack of histone acetylation due to histone deacetylase (HDAC) overexpression, and histone H3 lysine 27 trimethylation (H3K27me3) by polycomb repressive complex 2 (PRC2). It may also be due to inhibition of transcription factors for HLA class I APM components, by downregulation of NLRC5 that acts as a transcriptional activator of major histocompatibility complex (MHC) class I gene and interferon regulatory factor 1 (IRF1). In addition, HLA I expression is regulated by MAPK pathway including activation via EGFR, HER2, RAS, RAF, ALK or RET mutations/overexpression resulting in STAT1 inactivation. Lysosomal degradation of HLA class I heavy chain-β2m-peptide trimolecular complexes may also result in HLA class I downregulation on malignant cells, with autophagy resulting in expression of cargo receptor NBR1 involved in trafficking of trimolecular complexes to the lysosome and overexpression of PCSK9, a secreted protein binding to an extracellular region of HLA class I heavy chain that mediates endosomal-lysosomal degradation of trimolecular complexes.

Most HLA class I APM defects can be corrected by counteracting the abnormalities in epigenetic and/or regulatory mechanisms. Abnormalities in the expression, regulation and/or function of components of this machinery have been associated with the development of resistances to T cell-based immunotherapies. Restoring sensitivity to checkpoint inhibition may be achieved by using targeted strategies to enhance HLA class I expression. Restoration of HLA class I APM component expression in Merkel cell carcinoma cells by treatment with HDAC inhibitors in vitro provided the rationale for combining this approach with immune checkpoint inhibition. In a patient with Merkel cell carcinoma with complete loss of HLA class I expression and resistance to checkpoint inhibition, treatment with the HDAC inhibitor panobinostat restored HLA class I expression, increased CD8+ T cell infiltration, and resulted in disease stabilization following anti-PD-L1 treatment with avelumab.

Tumor cell-derived exosomes can also contribute to immune-cell dysfunction in cancer. Both normal cells and tumor cells in the TME produce exosomes, which are mixed populations of normal cell-derived and tumor cell-derived vesicles. The tumor antigen chondroitin sulfate proteoglycan 4 (CSPG4) can be used as a marker to separate exosomes released by cancer cells from exosomes released by non-malignant cells. Melanoma cell-derived exosomes inhibited C-type lectin CD69 expression, induced apoptosis, suppressed proliferation in CD8+ T cells and downregulated activating receptor NKG2D expression in NK cells while non-melanoma cell derived exosomes were enriched in immunostimulatory proteins [20]. Melanoma cell-derived exosomes may be a major mechanism of tumor-induced immune suppression and as a barrier to immunotherapy.

### Electrochemotherapy in metastatic melanoma

Electrochemotherapy (ECT) involves the application of high intensity electric pulses which increase the permeability of cell membranes, allowing the direct diffusion of cytotoxic drugs into cells. In addition to its use in skin cancers, ECT can be employed for the treatment of deep-seated lesions, including in the intestinal tract, pancreas, liver, and bone.

ECT can provide a durable benefit in melanoma as shown in the study of 60 patients that reported 48% had a complete response, that was long-lasting after one ECT session, and 13 patients (45% of complete responders) disease-free after a mean duration of follow-up of 27.5 months [21]. Similarly, a larger multicenter study, reported 67% 1-year OS and 74% melanoma-specific survival, indicating that ECT is a highly effective local treatment for melanoma metastases in the skin [22]. Coverage of deep margins, previous irradiation of the treated area and tumor size (< 3 cm) were all significantly associated with complete response to ECT. More recently, 11-year data from 28 centers across Europe that included 987 patients with 2482 tumor lesions were analyzed [23]. ORR was 85%, with 70% complete responses and 15% partial responses. Response rates were high across different histotypes, including metastases of malignant melanoma (82%), basal cell carcinoma (96%), breast cancer metastases (77%), squamous cell carcinoma (80%) and Kaposi's sarcoma (98%). Higher response rates were achieved with lesions < 3 cm. Hexagonal electrodes were generally used for larger tumors, but linear array electrodes provided better tumor control for tumors < 3 cm. Intravenous administration of bleomycin was more effective than intratumoral injection in tumors > 2 cm in size.

As with radiotherapy, ECT may induce an abscopal effect which suggests the potential for combining with immunotherapy. This may be due to the release of tumor antigens stimulating inflammatory response, cytokine production, complement activation, increased MHC class I expression and T cell activation as a result of checkpoint inhibition. In 15 patients treated with ipilimumab who underwent ECT for local disease control and/or palliation of cutaneous lesions, a local objective response was observed in 67% of patients (27% complete response) [24]. According to immune-related response criteria, a systemic response was observed in nine patients, resulting in a disease control rate of 60%. Response was associated with a significant decrease in levels of circulating Tregs. In another study of 127 melanoma patients treated with ipilimumab, the addition of local peripheral treatment (local irradiation, skin directed ECT or selective internal radiotherapy of liver metastases) significantly prolonged OS [25].

A randomized study to compare ECT with wide excision in patients with local cutaneous melanoma undergoing sentinel lymph node biopsy is planned.

## Mechanisms of resistance and drivers of response—session

### Translational research in the metastatic melanoma

Melanoma is a very heterogeneous disease driven by molecular alterations in oncogenic signaling pathways such as MAPK and phosphatidylinositol-3-kinase (PI3K). Many melanomas share features of the benign counterpart of the melanocytic skin lesions (melanocytic naevi). For example, molecular characteristics of a benign naevi bleu overlap with ocular melanoma. Congenital naevi typically have NRAS mutations while acquired naevi have BRAF mutations.

Genetic alterations are useful biomarkers for targeted therapy selection, but accumulating evidence strongly suggests that the pathogenesis of melanoma is also shaped by other factors. For example, high TMB alone or in combination with high IFN-γ gene expression signature have shown to be predictive of prolonged relapse-free survival (RFS) to adjuvant dabrafenib plus trametinib [26]. Of interest, among patients with high TMB but low IFN-γ gene expression signature, the benefit of targeted therapy was less prolonged with a sharp decrease in RFS after treatment discontinuation, which may imply longer duration of treatment is needed in this population.

Molecular alterations may be also important to understand the response to immunotherapy. For example, TMB and gene expression-based T cell-inflamed signature were independently predictive of a positive response to anti-PD-1 therapy and demonstrated low correlation, suggesting that they reflect distinct features [27].

Recently, integrative analysis of 2658 whole-cancer genomes and their matching normal tissues across 38 tumor types from the Pan-Cancer Analysis of Whole Genomes (PCAWG) Consortium of the International Cancer Genome Consortium (ICGC) and The Cancer Genome Atlas (TCGA) has been reported [28]. This revealed that the most common mutation overall was TP53. BRAF mutations comprise the most common genetic alteration in cutaneous melanoma and most common BRAF mutation is V600E, which represents 80% of alterations in the gene. Mutation V600E is associated with the superficial spreading subtype, younger patient age, and skin sites without chronic sun-induced damage (CSD). The V600K and V600R mutations are other known BRAF mutations. In contrast, V600K mutations are correlated with skin sites with CSD, such as the head and neck, and patients of older age. Overall, single-base-substitutions and doublet-base substitutions were shown to be those attributed to DNA damage induced by UV light, particularly enriched in these melanomas [29].

Accumulating evidence suggest that the pathogenesis and resistance to therapy of melanoma is also shaped by the aberrant activity of epigenetic factors that regulate gene expression through the modification of DNA chromatin structure regulators. The combination of DNA and histone modifications and DNA and histone binding proteins create an epigenetic code that controls genome-wide transcriptional networks. Hypermethylation affects key suppressor genes such as p16/INK4A. p14/ARF, RASSF1A and a CpG island methylator phenotype has been correlated with disease progression. In general, the interrelationship between genetic and epigenetic alterations of cancer cells has been known. A gene with a high frequency of mutation is less commonly changed by epigenetic alterations, a representative example of which is TP53.

**Fig. 1:**
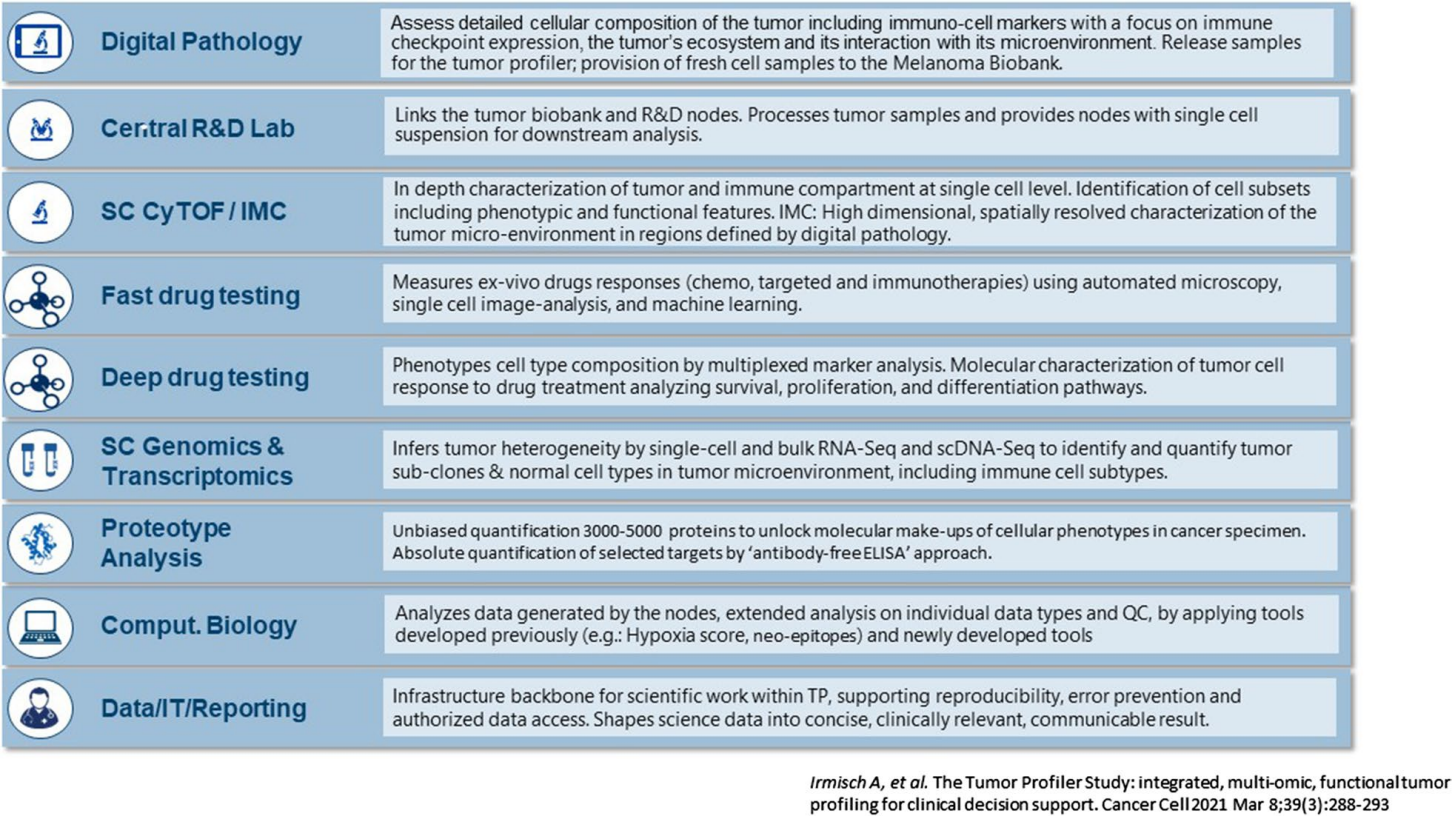
Multitude of cutting-edge technologies involved

A multitude of cutting-edge technologies developed to study tumor and TME. High-dimensional mass cytometry (CyTOF) allows the simultaneous measurement of multiple cellular markers at single-cell level. Using CyTOF, the frequency of CD14+ CD16+ HLA-DR hi monocytes was shown to be a strong predictor of progression-free and overall survival in response to anti-PD-1 immunotherapy was shown to be a strong [30]. CyTOF can also be applied to tissue imaging to evaluate status of the inflammation of melanoma metastases, e.g., quantify special interactions, T cell infiltration, potential of T cells to interact with PD-L1 expression or the presence of tertiary lymphoid structures. Analysis of the location of inflammatory cells in the lesion allows for spatial image analysis including proximity mode which can establish whether a tumor is cold or inflamed and help guide treatment decisions regarding the use of checkpoint inhibitors. The next step will be to utilize these types of investigations in a 3D setting. This and other techniques will provide a huge volume of data and high-performance medicine will require the convergence of human an artificial intelligence in order to develop new treatment algorithms (Fig. 1).

### Intrinsic tumor genomic and metabolic factors leading to immunoresistance

Tumors use various mechanisms to resist the immune system, the three main ones involving the avoidance of detection by downregulation of immune targets (e.g., through β2M loss, class I downregulation), the promotion of an inhibitory TME, or resisting death (e.g., the Pi3K pathway, glycolysis).

One protein produced by tumors is transforming growth factor (TGF)-β, a ubiquitous cytokine with pleiotropic effects on cell growth and differentiation which is tumor-suppressive in early cancer stages but immunosuppressive and tumor-promotional in later-stage disease. TGF-β limits immune responses to antigen presentation by inducing immune tolerance and inhibits the function and proliferation of T-cells. Tumors can be associated with very high levels of TGF-β, which has been shown to be elevated in the blood in patients with advanced stage cancer, including melanoma.

Tumor-infiltrating lymphocytes (TILs) can be engineered to express the TGF-β dominant negative receptor, which renders T cells resistant to TGF-β in the TME. In a small phase I trial, 8 of 12 heavily pretreated patients with metastatic melanoma had a response or stable disease following adoptive transfer of these engineered TILs, with some of these responses being durable.

Another way by which tumors can produce a negative environment and resist death is via protein arginine methyltransferases (PRMT), which are upregulated in many tumors. PRMT catalyzes the methylation of histones and non-histone proteins from *S*-adenosylmethionine (SAM) to arginine residues. Emerging data indicates that arginine methylation might be a driver for the initiation and progression of cancer. Nine members of the PRMT family have been identified and can be divided into types I, II and III enzymes. PRMT1, a member of the type I family, is the most abundant PRMT and is involved in transcription activation, signal transduction, RNA splicing and DNA repair. PRMT1 is overexpressed in breast cancer, bladder cancer, pediatric acute lymphoblastic leukemia and in NSCLC, upregulated in lung cancer and glioma tissue, and associated with poor prognosis of colon cancer. PRMT5 is involved in transcription repression, signal transduction and the piRNA pathway and is upregulated in lung, gastric, bladder, colon cancer and lymphoma, and overexpressed in breast cancer and epithelial ovarian cancer. Cytoplasmic expression is also associated with high-grade subtypes of primary lung adenocarcinomas. In cutaneous melanoma, upregulated expression of PRMT1 is associated with an immunologically cold phenotype and poor clinical outcomes. Type I PRMT inhibitor enhanced T cell mediated tumor killing both in vitro and in vivo and improved the antitumor activity of immune modulatory antibody in mice through increasing the infiltration of CD8 + T cells into tumor tissues.

### Achieving durable MAPK suppression and invigorating anti-melanoma T cell immunity

RAS mutant tumors have been shown to be largely refractory to inhibitors of the MAPK pathway. Type I RAF inhibitors (i.e., vemurafenib, dabrafenib, and encorafenib) specifically inhibit monomeric V600 BRAF mutants but cause paradoxical activation of the MAPK pathway in NRAS mutant and/or dimeric RAF-active melanoma. The combination of type I RAF inhibitor plus MEK inhibitor suppresses acquired resistance in tumors driven by BRAF V600 mutations, although MAPK pathway reactivation still occurs. In contrast, type II RAF inhibitors inhibit monomeric and dimeric V600 BRAF mutants and CRAF/BRAF or CRAF/CRAF dimers with equal potency. As single agents, type II RAF inhibitors do not appear to be highly active. However, they may potentially be combined with allosteric MEK inhibitors to prevent the development of resistance.

In NRAS mutant melanoma PDX models, responses to daily treatment with the type II RAF inhibitor BGB-283 or the MEK inhibitor trametinib alone were limited. However, the combination of type II RAF inhibitor with trametinib achieved highly durable tumor regression in all tumors [31]. This was through stabilizing p-MEK in RAF complexes, leading to reduced MEK dimerization and uncoupling of MEK or p-MEK interaction with ERK.

High mutational burden in both BRAF and NRAS mutant syngeneic mouse models prolonged MEK inhibitor responses in a CD8 T cell-dependent manner. Systemic CD8+ T cell neutralization negated the increase in durability but had no effect on the development of MEK inhibitor resistance in lower mutational burden tumors. This indicates that CD8+ T cells suppress acquired resistance to MEK inhibitors through neoantigen recognition. Using multiple syngeneic or immune-competent tumor models, including NRAS mutant melanoma, KRAS mutant pancreatic ductal adenocarcinoma and KRAS mutant colorectal cancer, type II RAF inhibitor plus trametinib synergistically suppressed resistance and were able to induce tumor regression in well-established tumors in combination but not alone. In vivo response to trametinib plus type II RAF inhibitor was dependent on CD8+ T cells. Trametinib alone resulted in a reduction in large T cell clones over time, while trametinib plus type II RAF inhibitor prevented this loss and promoted clonal expansion and intratumorally elicited PD-1/Ki-67-high CD8+ T cells. Anti-PD-L1 therapy further enhanced durability of response to trametinib plus type II RAF inhibitor in an NRAS mutant melanoma model, consistent with systemic and/or intratumoral CD8+ T cells being an important effector of type II RAF inhibition plus MEK inhibition in immunocompetent hosts. Combination trials of type II RAF inhibitor plus MEK inhibitor plus anti-PD-1/L1 therapy may be warranted in RAS/MAPK-hyperactivated cancers.

### Tumor mutation burden and liquid biopsies

TMB, which is defined as the number of somatic single nucleotide variants, InDel- and essential splicing changes in the complete coding region (exome) and reported as mutations per mega base, has been suggested as a predictive biomarker for response to checkpoint inhibitor therapy. In a study of 35 patients with melanoma treated with ipilimumab and nivolumab, TMB was significantly higher in responders than in non-responders and TMB-high status (> 23.1 Mut/Mb) was associated with a survival benefit versus TMB-low or TMB-intermediate (TMB ≤ 23.1 Mut/Mb) [32]. Several retrospective studies across different tumor types, but in particular melanoma and NSCLC, have also reported that higher TMB is correlated with improved response rates and survival times with immune checkpoint inhibitor therapy [33]. A positive correlation between TMB and improved response to checkpoint blockade is not unexpected, given the higher the mutational load the higher the probability that neoantigens are presented on the tumor surface. However, TMB is not a prognostic factor in thick primary cutaneous melanoma (unpublished data).

Another potential predictive biomarker is circulating tumor DNA (ctDNA). In the melanoma prospective biomarker study, repetitive liquid biopsies were taken, cell-free DNA was extracted and at least one driver mutation was monitored in each patient using digital droplet polymerase chain reaction from ctDNA [32]. Non-detectable ctDNA at baseline was a positive predictive marker for response to immune checkpoint inhibitors and was associated with improved survival. Increasing ctDNA was almost only observed in progressive patients, with no increase in ctDNA during therapy also a positive predictive marker for the efficacy of immune checkpoint inhibitors. ctDNA remaining or becoming undetectable at first follow-up after 3 weeks was significantly more common in responders, suggesting the potential of accurately predicting treatment early after starting treatment. Other studies have also shown that longitudinal assessment of ctDNA in patients with metastatic melanoma treated with PD-1 inhibitors is an accurate predictor of response to therapy, PFS and OS [34]. The S100 serum protein has been the gold standard for monitoring of melanoma tumor progression. In an analysis of 115 plasma samples from 47 melanoma patients, ctDNA was shown to correlate with S100 and be at least as effective as S100 in predicting response to changes in tumor load.

### Mechanisms of resistance to cancer immunotherapy

The T cell-inflamed and non-T cell-inflamed TME represent two categories of immune escape. T cell inflamed tumors are characterized by the presence of CD8+ T cells, expression of a chemokine signature that recruits T cells into the TME and a type 1 IFN signature that suggests innate immune pathways necessary to generate a spontaneous T cell response. These tumors are characterized by inhibitory regulatory pathways that allow immune escape. In contrast, non-T cell-inflamed tumors lack this inflammatory signature with an absence of intratumoral CD8+ cells and immune escape mediated by T cell exclusion. Most patients who respond to immunotherapy have T cell-inflamed tumors, with activity of anti-PD-1 agents associated with a T cell inflamed signature at baseline across multiple cancers. Primary resistance to checkpoint blockade is most often due to a non-T cell inflamed TME.

Understanding the factors that regulate the degree of spontaneous T cell infiltration is critical to understand PD-1 resistance and response to immunotherapy. These may be tumor-intrinsic oncogenic events, environmental differences (e.g., commensal microbiota), or germline polymorphisms in immune regulatory genes. Secondary resistance to immunotherapy may also be associated with tumor cell-intrinsic immune-evasive oncogenic alterations. For example, secondary resistance to immunotherapy in melanoma was associated with loss of immune signature and either upregulated β-catenin or PTEN deletion in two patients, suggesting selection for new oncogenic variants that mediate T cell exclusion [35].

In vitro CRISPR screening identified 2,4-dienoyl-CoA reductase 2 (DECR2) as an immunotherapy resistance gene and, in a mouse model, DECR2 knock-down tumors were resistant to PD-1 blockade [36]. DEC2R encodes a peroxisomal 2,4-dienoyl-CoA reductase and is involved in intracellular lipid metabolism and has a role in a cell death pathway distinct from apoptosis known as ferroptosis. Immunotherapy-activated CD8+ cells induce ferroptosis of tumor cells [37]. DEC2R knockdown cells have altered expression of ferroptosis-related genes and DEC2R knockdown tumor cells are relatively resistant to ferroptosis inducers in vitro. DEC2R knockdown tumor cells also fail to generate oxidized lipids in response to CD8+ T cells. In melanoma samples, anti-PD-1 clinical benefit is associated with increased expression of DEC2R and decreased expression of a component of several heterodimeric amino acid transporter complexes SLC3A2. These data suggest tumor cell ferroptosis may be a major mechanism for T cell mediated tumor cell killing and may be a target pathway for improving immunotherapy efficacy.

### T-cell intrinsic mechanisms of resistance to PD1 checkpoint blockade

T cell related resistance mechanisms may explain why some but not all patients respond to PD-1 checkpoint blockade. T cell costimulatory receptor CD28 that provides secondary signal for T-cell activation is a primary target for PD-1 mediated inhibition and rescue of exhausted CD28 T cells by PD-1 inhibitors is CD28 dependent, suggesting that PD-1 directly effects the T cell signaling pathway.

Phospho-proteomics and flow cytometry-based analysis of patient-derived T cells from responders and non-responders to PD1 inhibitors identified mediators, signaling components and pathways associated with PD-1 checkpoint blockade resistance. Co-expression of T cell inhibitory receptors (BTLA and TIM3) with CD28 and PD-L1 was higher in patients with melanoma who were resistant to anti-PD-1 therapy suggesting that these receptors might have negative impact on melanoma patients outcome and be predictive biomarkers of resistance to anti-PD1 pathways blockade.

BTLA and TIM3 co-expression was associated with resistance only in patients with CD28+ T cells. Differential expression of tyrosine phosphatase pShp-2 and tyrosine kinase Src pY418 was shown in PD-1 positive T cell subsets indicating that pShp-2 effectively antagonizes Src-dependent phosphorylation and negatively regulates signal transduction pathway in T cells. Also, T cell proliferation did not substantially change after removal of PD-L1 in T cells from resistant patients, whereas there was increased proliferation in patients responding to anti-PD1 therapy. This indicates that the level of PD-L1+ cells was strongly correlated with a number of markers indicating a negative immune response.

It has been proposed that PD-1 suppresses initial signaling following T cell receptor (TCR) ligation, which inhibits upregulation of CD8 binding to the TCR-pMHC complex and blocks further T cell activation enhancement. Melanoma impaired TCR 2D affinity in a tissue-restricted fashion in a mouse model, with reduced TCR-pMHC-CD8 binding in tumor and tumor-draining lymph node samples, indicating that binding is somehow impaired in the TME. Blocking the PD-1/PD-L1 interaction increased the 2D affinity of TCR-pMHC interactions, with anti-PD-1 or anti-PD-L1 therapy overcoming the negative effect of the TME on TCR-pMHC-CD8 affinity.

CD8-pMHC binding and Lck activity are required for upregulated CD8 binding to prebound TCR-pMHC complex. Biomembrane Force Probe measurements show that the activities of TCR-proximal signaling components affect T-cell mechanosensing and sensitivity at the earliest stages of antigen recognition. This step in TCR signaling is influenced by PD-1 and other inhibitory receptors via Shp-1/2 targeting CD28 and Lck to directly suppress TCR-pMHC-CD8 binding. TCR-pMHC binding was independent of PD-1-PD-L1 interaction, but TCR-pMHC-CD8 binding was suppressed by PD-1 or PD-L1, demonstrating negative cooperativity as fewer bonds formed than the sum of bonds formed by each interaction alone.

**Fig. 2:**
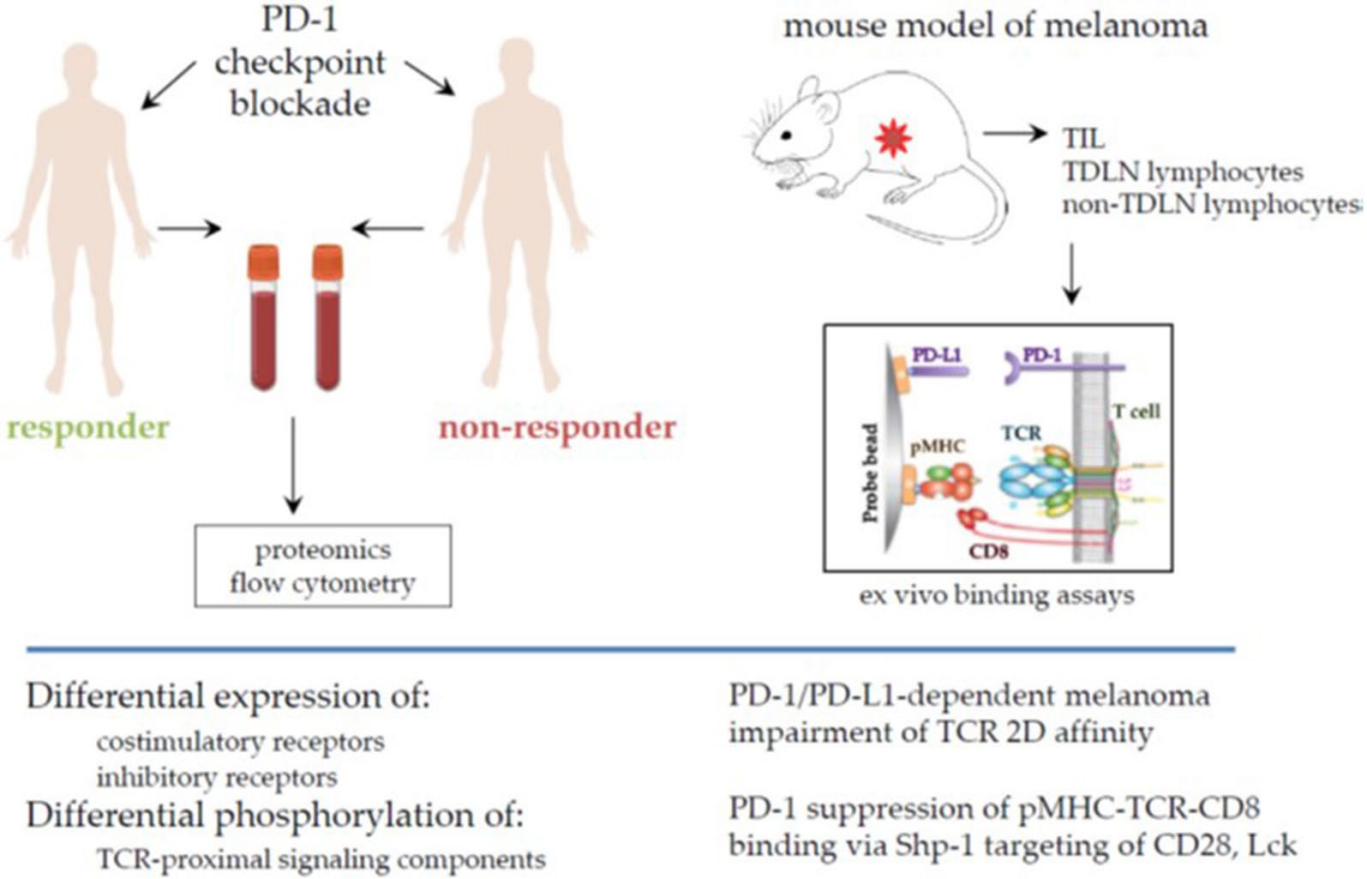
T-cell intrinsic mechanisms of resistance to PD1 checkpoint blockade

These data suggest that targeting these interactions and understanding how PD-1 signaling impacts T cell sensitivity may be important for identifying new molecular targets to enable T-cells to overcome dysfunction during PD-1 checkpoint blockade and enhance the response of PD-1-high cells to checkpoint blockade (Fig. 2).

### Targeting dendritic cells to enhance the effectiveness of PD1 blockade against cold tumors

Complementary targeting of the induction and effector phases of cancer immunity may be an effective approach in improving the effectiveness of treatments. Dendritic cells (DCs) are potent antigen-presenting cells that present tumor-specific peptides for T cell activation. Induction and expansion of CTLs with high expression of the effector-type chemokine receptors CXCR3 and CCR5 can be achieved through the use of α-type-1-polarized DC (αDC1) vaccines matured in the presence of type-1 and type-2 interferons. Chemokine modulation, e.g. with IFN-α, toll-like receptor (TLR)-3 agonist and celecoxib, can reverse an immunosuppressive chemokine profile and increase the trafficking of CTLs into tumors. The cytokine microenvironment regulates the expression of various T-cell subtypes and is the rationale for combining chemokine modulation with DC vaccines, with the vaccine expected to induce tumor-specific CTLs, and chemokine modulation expected to direct CTLs to the tumor. In theory, once CTLs are in the tumor, their survival and killer function can be improved through immune checkpoint inhibition.

IL-12 production by DCs is needed for the induction of tumor-specific CTLs ex vivo, and the activation of NK cells and Th1 cell induction. DC-produced IL-12 predicts prolonged PFS and/or OS in DC-vaccinated patients with advanced glioblastoma [38], melanoma [39] and other cancers [40, 41]. Autologous αDC1-induced melanoma-specific CTLs are highly cytolytic and express high levels of chemokine receptors CXCR3 and CCR5 [42]. Preclinical and early clinical data demonstrate that the chemokine-modulating regimen targeting TLR-3, type 1 IFN and the prostaglandin E2 system, selectively enhance CTL numbers but reduce Tregs in the TME, thereby uniformly sensitizing tumors for the therapeutic effectiveness of PD-1 blockers and DC vaccines. This is being further evaluated in patients with melanoma, colorectal cancer, and ovarian cancer in a new NCI-funded program project (P01). The combination of an αDC1 vaccine (αDC1 loaded with tumor blood vessel-targeting antigenic peptides [43]) or with desatinib or with chemokine modulation followed by anti-PD-1 therapy are being investigated in melanoma patients with primary PD-1 resistance (NCT01876212 and NCT04093323).

## Emergent strategies—session

### Neoadjuvant immunotherapy—less is more

In preclinical models, neoadjuvant immunotherapy has been shown to activate a broader and more diverse range of tumor-resident T cell clones compared with adjuvant treatment, thereby inducing a broader immune response. For example, in the OpACIN trial, neoadjuvant treatment with ipilimumab plus nivolumab resulted in expanded more tumor-resident T cell clones than when given as adjuvant therapy [44]. After a median follow-up of 4 years, none of the seven patients with a pathologic response in the neoadjuvant arm had relapsed [45]. Estimated 4-year RFS rate was 60% in both the neoadjuvant and adjuvant arms while, the 4-year OS rates were 90% and 70%, respectively.

The toxicity of the neoadjuvant ipilimumab plus nivolumab dosing schedule in the OpACIN trial was significant and so the OpACIN-neo trial was designed to identify an equally effective but more tolerable dosing schedule of ipilimumab plus nivolumab. This was an open-label trial in which patients with resectable stage III melanoma were randomized to one of three different ipilimumab plus nivolumab dosing regimens [46]. Of these, two cycles of ipilimumab 1 mg/kg plus nivolumab 3 mg/kg every 3 weeks was identified as tolerable and effective, inducing a pathological response in 77% of patients. At 2-year follow-up, almost all high-grade adverse events had resolved to ≤ grade 1, except for grade 2 endocrinopathies. More than 80% of patients were relapse-free at 2-years without adjuvant treatment, and RFS remained significantly higher for patients with pathologic response versus non-responders (97% versus 36%). Only one of 64 patients with a pathological response had relapsed. Pathologic response appears to be the strongest predictor for RFS. However, high baseline TMB and IFN-γ signature expression might also predict RFS and help identify patients that would benefit from neoadjuvant ipilimumab plus nivolumab [46].

PRADO is an extension cohort of the OpACIN-neo study, one aim of which is to assess personalized response-driven adjuvant therapy. At a minimum of 12 weeks follow-up, 70 of 99 patients (71%) had a pathological response in the index lymph node [47]. Sixty patients (61%) had major pathologic response in the index lymph node and did not undergo therapeutic lymph node dissection. These patients had fewer surgery-related adverse events and higher quality-of-life scores.

Neoadjuvant therapy is an active area of research with numerous ongoing trials assessing different immunotherapy combinations. The International Neoadjuvant Melanoma Consortium is developing recommendations to ensure alignment of trial designs and correlative analyses across many of these studies [48].

### What are new ways to think about immunotherapy combinations for melanoma?

When we attempt to address the question of whether combination immunotherapy is better than single agent therapy, the typical approach is to assess whether response or survival are improved by treatments in combination as compared to single agents. However, there are other ways to think about how to develop combinations in melanoma. These include the possible de-escalation of combinations, in which components of the combination are discontinued at certain times based on clinical events (response or toxicity) and treatment is continued as monotherapy. In this scenario, clinical trials may be initiated with several drugs in combination, with some stopped or substituted and treatment adapted over time, depending on initial responsiveness.

Nivolumab and ipilimumab is the most investigated immunotherapy combination, with 5-year OS data available [49]. Lessons learned from this combination may be relevant to the development of future combinations with newer drugs. With nivolumab and ipilimumab, survival outcomes appear to be similar even among patients who discontinue treatment early because of adverse events [49, 50]. Similarly, in the adjuvant setting after resection or radiation of stage IV melanoma, nivolumab plus ipilimumab increased RFS compared with placebo even though patients only received a median of two doses of the combination [51]. This suggests that a short duration of combination therapy may be as effective as longer treatment, especially in the context of a high level of side effects.

Following these data, a logical question arose; what about stopping combination immunotherapy after initial treatment benefit? This question was prospectively assessed in the Adaptively Dosed ImmunoTherapy (Adapt-IT) study, in which 60 patients with unresectable stage III/IV melanoma received two doses of nivolumab 1 mg/kg plus ipilimumab 3 mg/kg before an interim CT scan at 6-weeks [52]. Patients with a response (or stable disease without an increase in total measurable tumor burden) were switched to nivolumab alone while patients with an increase in tumor burden continued with the standard third and fourth doses of the nivolumab + ipilimumab combination before switching to nivolumab. Over two-thirds of patients (68%) had tumor shrinkage or no growth at week 6 after one or two doses of the combination. The best ORR of 58% achieved in this trial appears comparable to that achieved with standard nivolumab plus ipilimumab dosing. Immunologic effects in blood occurred after the first dose and did not further increase after the second dose, so it may be that efficacy can be driven by a single dose of nivolumab + ipilimumab and a trial to investigate a single combination dose is under development.

Moving forward, a de-escalation of treatment approach may be employed together with the possible addition of different treatments to patients who do not respond favorably after two doses of anti-PD-1 and anti-CTLA-4 in combination. CD8 T cell imaging, circulating tumor DNA, or peripheral blood markers of immune activation may be used in the future to inform when treatment might be escalated or de-escalated.

### Considering QUAD therapy to address unmet needs in high risk BRAF mutant melanoma

Two randomized trials have reported on triplet combination of BRAF plus MEK inhibition with PD-1/PD-L1 therapy. In the IMSPIRE-150 trial, atezolizumab in combination with vemurafenib and cobimetinib resulted in a significant improvement in PFS versus vemurafenib and cobimetinib without atezolizumab (15.1 vs 10.6 months; HR: 0.78; p = 0.025) [53]. There was also a suggestion of improved OS with the triplet combination. These results are comparable with those of the COMBI-I trial, in which combined dabrafenib and trametinib with spartalizumab led to an improvement in the primary endpoint of median PFS compared with dabrafenib and trametinib plus placebo [54]. However, unlike in IMSPIRE-150, this did not reach statistical significance (16.2 versus 12.0 months; HR: 0.820, p = 0.042). Toxicity from the triplet regimens was substantial in both these studies, with over 70% of patients having grade ≥ 3 treatment-related adverse events. However, most were events known to occur with targeted therapy and management of these events is feasible. Thus, the value of the triplet approach to the general melanoma patient population remains unclear.

Compared with other landmark trials of targeted and immunotherapy that underpin the current standard of care, the PFS seen with the triplet combinations in these trials was generally favorable. However, OS data were not impressive compared to that seen in other trials, even of PD-1 monotherapy, and do not appear to be comparable with OS achieved with nivolumab plus ipilimumab. Thus, although BRAF plus MEK inhibition and anti-PD-1 plus anti-CTLA-4 are backbone therapies in melanoma, the BRAK/MEK/PD-1 triplet combination has not progressed the treatment landscape.

Patients with a high TMB or T cell-inflamed gene expression profile are more likely to respond to immunotherapy [27]. Similarly, in the safety run-in and biomarker cohorts of the COMBI-I trial, patients with shorter PFS were those with low TMB and low T cell-inflamed gene expression profile [55]. This suggests the triplet regimen may augment responses in those patients already likely to respond.

The optimal use of targeted and immunotherapy remains an open question. One possibility is that a more nuanced sequential use of targeted and immunotherapy may be more beneficial than the type of upfront triplet combination approach investigated in IMSPIRE-150 and COMBI-I. For instance, early data from the SECOMBIT trial has suggested a ‘sandwich’ approach of 8 weeks of targeted therapy (encorafenib plus binimetinib) followed by immunotherapy (nivolumab and ipilimumab) until disease progression before switching back to targeted therapy may be promising [56].

There is still an unmet need for those 50% of patients who do not achieve 5-year OS on combined immunotherapy, with existing therapies seeming to benefit the same subset of patients. One consideration is that MEK inhibition may be having a deleterious role in the combination by dampening T cell activation and reconsideration of its role in targeted and immunotherapy combinations is needed. One attempt to answer this is the planned QUAD study which will include two dose cohorts: triplet therapy of BRAF inhibitor plus anti-PD-1 and low-dose anti-CTLA-4 and quadruple therapy of combined BRAF inhibitor, MEK inhibitor, anti-PD-1 and anti-CTLA-4. One of these regimens will then be assessed in higher-risk patients (e.g., with symptomatic brain metastases, elevated LDH with liver metastases, or sum of longest diameters of target lesions > 44 mm).

### Development of novel antibodies targeting regulatory T cells in cancer

CD25 is the high affinity subunit of the IL-2 receptor and is highly expressed on Tregs and transiently upregulated on activated T effector cells (Teffs) in vitro. In murine tumors, CD25 is primarily restricted to Tregs with minimal expression on Teff cells. In humans, CD25 is preferentially expressed on tumor infiltrating Foxp3+ Treg cells. Similar to mouse models, CD25 expression was significantly higher on CD4+FoxP3+ Treg cells relative to CD4+FoxP3− and CD8+ T cells within the studied tumor subtypes of advanced melanoma, early NSCLC, and renal cell carcinoma.

However, CD25 was largely dropped as a therapeutic target. In mice, anti-CD25 as an intervention against established tumors failed to delay tumor growth or prolong survival and did not enhance the activity of immunotherapies. This has been attributed to ineffective intratumoral Treg cell depletion due to upregulation of the inhibitory Fcγ receptor IIb at the tumor site. Use of an anti-CD25 antibody engineered with enhanced binding to activating Fcγ receptors and antibody-dependent cell-mediated cytotoxicity (ADCC) led to effective depletion of tumor-infiltrating Tregs and increased Teff to Treg cell ratios [57]. Fc-optimized anti-CD25 also synergized with anti-PD-1 to reject established tumors with no evidence of immune-related toxicity.

Clinical anti-CD25 antibodies (daclizumab, basiliximab) block the IL-2/IL-2R interaction, as does the αCD25-m2a antibody (PC61 clone). However, bystander IL-2 receptor signaling blockade on non-depleted effector T cells limits their antitumor activity. A depleting/non-IL-2 blocking anti-CD25 antibody could in theory be more effective against tumors. A next-generation anti-CD25 antibody optimized to deplete Treg cells while preserving IL-2-STAT5 signaling on effector T cells has shown potent single dose activity, which is dependent on the ability of effector cells to sense endogenous IL-2 [58].

The first non-IL-2 blocking anti-human CD25 antibody to be developed is RG6292. This targets CD25 high Tregs without interfering with IL-2 signaling while afucosylated human IgG1 mediates enhanced ADCC. RG6292 preferentially depletes Tregs in peripheral blood mononuclear cells and human tumor samples. RG6292 also depletes Tregs and activate effector cells in tumors from humanized mice. A phase 1 study of RG6292 is ongoing to assess the safety, efficacy, pharmacodynamics, and pharmacokinetics in patients with advanced solid tumors (NCT04158583).

### Oncolytic virus combination with checkpoint inhibition

Many viruses have been investigated as anticancer agents based on their ability to induce oncolysis. T-VEC is an oncolytic virus derived from the Herpes Simplex Virus-1 (HSV-1) strain JS1. Several genetic modifications have been made to T‐VEC to enhance tumor cell selectivity, restore antigen presentation, and enhance immune recognition of HSV‐infected tumor cells while minimizing toxicity. Because of its favorable safety profile and oncolytic properties, T‐VEC is a candidate for use in combination with checkpoint‐blockade.

T-VEC is being assessed in combination with ipilimumab versus ipilimumab alone for advanced melanoma. In the primary analysis, conducted approximately 6 months after the last patient was enrolled, the ORR was significantly higher in T‑VEC plus ipilimumab treated patients versus ipilimumab alone (39% vs 18%; odds ratio, 2.9; 95% CI 1.5–5.5; P = 0.002) [59]. Moreover, responses were not limited to injected lesions as decrease in visceral lesions was observed in 52% of patients in the combination arm and 23% in the ipilimumab arm. Combination treatment was tolerable and not associated with unexpected adverse events or an increase in the incidence or severity of adverse events for either agent. At 4-year follow-up, responses were durable and had remained stable between 3 and 4 years. PFS trended in favor of the combination arm but was not significantly different (HR, 0.81; 95% CI 0.54–1.25, P = 0.23). Median OS and median duration of response had not been reached. Patients treated with ipilimumab alone were more likely to receive subsequent anticancer therapy and to receive the therapy earlier, which may confound OS analysis. In a *post-hoc* BRAF V600 mutation subgroup analysis, there was a numerical PFS improvement for the wild-type subgroup, an observation worthy of further investigation. No additional safety signals were observed in longer-term follow-up.

In the phase Ib single-arm part of the trial, the combination of T‐VEC plus pembrolizumab resulted in an ORR of 62% with a complete response rate of 33%, and no dose‐limiting toxicities were observed [60]. In a follow‐up efficacy analysis of the phase Ib part after a median follow‐up time of 36.8 months, ORR was 67%, Median OS was not estimable, and 3-year OS rate was 71.4%.

T-VEC has been combined with pembrolizumab in the pivotal phase Ib/IIIMASTERKEY‐265 trial. However, the trial has recently been stopped for futility, necessitating our rethinking of further development of oncolytic viruses, including biomarkers for better patient selection.

### Exploring the potential of dendritic cell therapy for the treatment of advanced melanoma from monocyte dendritic cells to myeloid dendritic cells

DCs can initiate and direct adaptive immune responses. In DC vaccination, DCs are educated ex vivo to present tumor antigens and are administered to the patient with the aim of inducing a tumor-specific immune response. However, monocyte-derived DCs (moDCs) may not be the best source for DC-based immunotherapy, due to decreased migratory capacities towards the site of T-cell interaction by exhaustion of the cells, and evidence of clinical antitumor activity with moDC vaccination is limited.

Synthetic mRNA electroporated moDCs (TriMixDC-MEL) administered by intravenous administration showed improved antitumor activity with durable disease control in patients with advanced melanoma [61]. Similarly, data from an adjuvant trial in patients with stage III/IV melanoma showed that TriMixDC-MEL was tolerable and may offer protection against disease recurrence, with an improvement in median time to non-salvageable recurrence [62]. Another phase II study investigated TriMixDC-MEL in combination with ipilimumab in patients with pretreated advanced melanoma. Six-month disease control rate was 51% and the ORR was 38%. These responses were durable, with seven complete responses and one partial tumor response ongoing after a median follow-up of 36 months [63].

Myeloid DCs (myDCs) have been shown to have a pivotal role in initiating antigen-specific antitumoral immunity. They are essential for priming antitumor T-cell responses and for relicensing antitumor T lymphocytes to eradicate tumor cells within the TME. Exclusion of myDCs from the TME may also be a tumor-intrinsic mechanism of immune evasion. MyDCs may also synergize with checkpoint inhibitor therapy. Anti-PD-1 checkpoint blockade requires the crosstalk between T cells and DCs, in particular cDC1 [64]. Human myDCs exist in two subsets that are differentiated by expression of either the BDCA-1 or BDCA-3 surface marker. In the myDAvlpNi trial, patients with advanced cancers received intratumoral combinatorial administration of unmanipulated CD1c (BDCA-1+) myDCs plus ipilimumab and avelumab in combination with intravenous low-dose nivolumab [65]. Tolerability was manageable with mainly injection-site reactions, but systemic immune-related adverse events were observed (pneumonitis, colitis, and bullous pemphigoid). Responses were seen in injected lesions but with little indication of activity elsewhere, other than in one patient with melanoma.

**Fig. 3:**
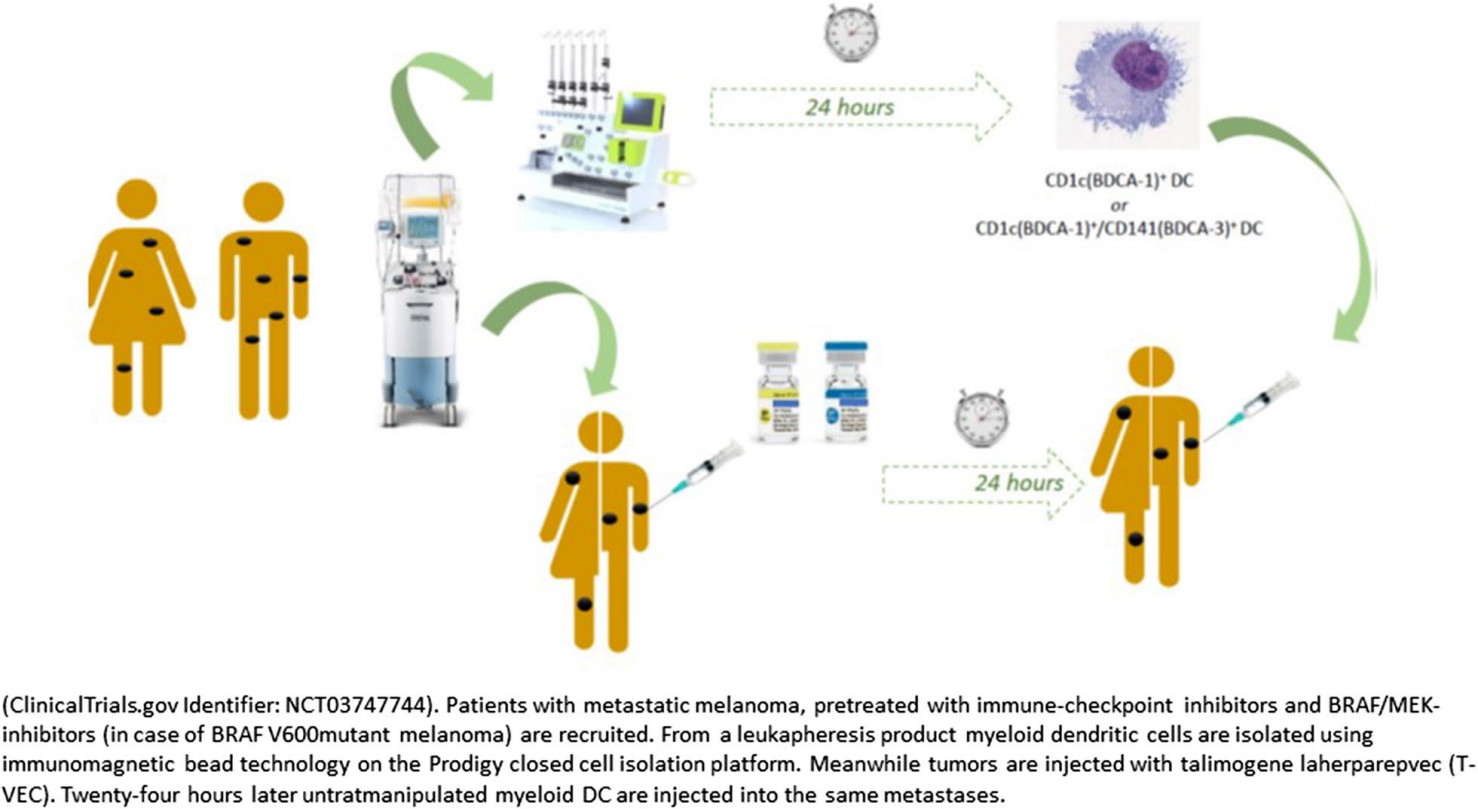
Schematic outline of the myDCTV phase I clinical trial

A cell product including both BDCA-1+ and BDCA3+ myDCs was then developed and assessed in combination with T-VEC. In the myTVDC trial, 12 patients were treated with intratumoral TVEC and CD1c (either BDCA-1+ alone or BDCA-1+ and BDCA-3+) myDCs [66]. Treatment appeared to be feasible and tolerable and resulted in signs of antitumor activity in injected as well as non-injected lesions. Based on preliminary data from these ongoing trials, intratumoral injection of myDCs with other immunotherapies is associated with manageable toxicity. These and other trials in different tumor types and associated translational research may provide better insight into the biology of myDCs and open new avenues for combinatorial treatment strategies (Fig. 3).

## Conclusions

Advances in molecular targeted therapy and immune checkpoint inhibition have led to unprecedented improvement in overall survival for patients with advanced melanoma. The use of immunotherapies, especially immune checkpoint inhibitors including PD1/PD-L1 and CTLA-4, as well as antagonists or agonists of other T cells immune regulatory receptors, and combinations with targeted BRAF and MEK inhibitors and novel agents such as oncolytic viruses have significantly improved outcomes for many patients with metastatic melanoma. Increased use of systemic treatments in the neoadjuvant and adjuvant settings may also help improve long-term outcomes for patients.

Despite these improvements, additional therapeutic approaches are needed to overcome resistance, develop novel biomarker strategies, and overall to advance precision medicine. Better understanding of the TME and host immune response may lead to development of biomarkers that help identify patients for the best treatment option, as well as new treatments and new combination strategies. Approaches to overcome resistance and to potentiate the immune response are being developed. Increasing evidence emerges that tissue and blood-based biomarkers can predict the response to a therapy. Predictive biomarkers can be considered for patients’ stratification or selection of patients who will most likely achieve favorable clinical outcome.

Here we reviewed approaches evaluating novel immunomodulatory agents and recent insights into genetic and phenotypic characterization of specimens from patients with melanoma to develop robust biomarkers to guide clinical decision-making.

## Data Availability

Not applicable.

## Abbreviations

ADCC: Antibody-dependent cell-mediated cytotoxicity
APM: Antigen-processing machinery
β2m: Beta 2 microglobulin
BMI: Body mass index
CSD: Chronic sun-induced damage
CSPG4: Chondroitin sulfate proteoglycan 4
ctDNA: Circulating tumor DNA
CTL: Cytotoxic T lymphocyte
CTLA-4: Cytotoxic T-lymphocyte-associated antigen
CyTOF: High-dimensional mass cytometry
DC: Dendritic cell
DECR2: 2,4-Dienoyl-CoA reductase 2
DRiP: Defective ribosomal product
ECT: Electrochemotherapy
FDA: Food and Drug Administration
FMT: Fecal microbiota transplant
HDAC: Histone deacetylase
HLA: Human leukocyte antigen
HSV-1: Herpes Simplex Virus-1
IFN: Interferon
IL-2: Interleukin-2
LDH: Lactate dehydrogenase
MAPK: Mitogen-activated protein kinase
MDSC: Myeloid-derived suppressor cell
MHC: Major histocompatibility complex
miRNA: Micro-RNA
moDC: Monocyte-derived dendritic cell
myDC: Myeloid dendritic cell
NK: Natural killer
NSCLC: Non-small-cell lung cancer
ORR: Objective response rate
OS: Overall survival
PD-1: Programmed death-1
PD-L1: Programmed death ligand-1
PFS: Progression-free survival
PI3K: Phosphatidylinositol-3-kinase
POM-1: Sodium polyoxotungstate
PRC2: Polycomb repressive complex 2
RFS: Relapse-free survival
SAM: *S*-Adenosylmethionine
SLiP: Short-lived protein
Teffs: T effector cells
TGF: Transforming growth factor
TIL: Tumor-infiltrating lymphocyte
TLR: Toll-like receptor
TMB: Tumor mutation burden
TME: Tumor microenvironment
TNF: Tumor necrosis factor
Tregs: Regulatory T cells
T-VEC: Talimogene laherparepvec
VGLL-1: Vestigial-like-1
